# A Dual Role Hypothesis of the Cortico-Basal-Ganglia Pathways: Opponency and Temporal Difference Through Dopamine and Adenosine

**DOI:** 10.3389/fncir.2018.00111

**Published:** 2019-01-07

**Authors:** Kenji Morita, Yasuo Kawaguchi

**Affiliations:** ^1^Physical and Health Education, Graduate School of Education, The University of Tokyo, Tokyo, Japan; ^2^International Research Center for Neurointelligence (WPI-IRCN), The University of Tokyo Institutes for Advanced Study, Tokyo, Japan; ^3^Division of Cerebral Circuitry, National Institute for Physiological Sciences, Okazaki, Japan; ^4^Department of Physiological Sciences, Graduate University for Advanced Studies, Okazaki, Japan

**Keywords:** reinforcement learning, reward prediction error, cost, basal ganglia, dopamine, adenosine

## Abstract

The hypothesis that the basal-ganglia direct and indirect pathways represent goodness (or benefit) and badness (or cost) of options, respectively, explains a wide range of phenomena. However, this hypothesis, named the Opponent Actor Learning (OpAL), still has limitations. Structurally, the OpAL model does not incorporate differentiation of the two types of cortical inputs to the basal-ganglia pathways received from intratelencephalic (IT) and pyramidal-tract (PT) neurons. Functionally, the OpAL model does not describe the temporal-difference (TD)-type reward-prediction-error (RPE), nor explains how RPE is calculated in the circuitry connecting to the DA neurons. In fact, there is a different hypothesis on the basal-ganglia pathways and DA, named the Cortico-Striatal-Temporal-Difference (CS-TD) model. The CS-TD model differentiates the IT and PT inputs, describes the TD-type RPE, and explains how TD-RPE is calculated. However, a critical difficulty in this model lies in its assumption that DA induces the same direction of plasticity in both direct and indirect pathways, which apparently contradicts the experimentally observed opposite effects of DA on these pathways. Here, we propose a new hypothesis that integrates the OpAL and CS-TD models. Specifically, we propose that the IT-basal-ganglia pathways represent goodness/badness of current options while the PT-indirect pathway represents the overall value of the previously chosen option, and both of these have influence on the DA neurons, through the basal-ganglia output, so that a variant of TD-RPE is calculated. A key assumption is that opposite directions of plasticity are induced upon phasic activation of DA neurons in the IT-indirect pathway and PT-indirect pathway because of different profiles of IT and PT inputs. Specifically, at PT→indirect-pathway-medium-spiny-neuron (iMSN) synapses, sustained glutamatergic inputs generate rich adenosine, which allosterically prevents DA-D2 receptor signaling and instead favors adenosine-A2A receptor signaling. Then, phasic DA-induced phasic adenosine, which reflects TD-RPE, causes long-term synaptic potentiation. In contrast, at IT→iMSN synapses where adenosine is scarce, phasic DA causes long-term synaptic depression via D2 receptor signaling. This new Opponency and Temporal-Difference (OTD) model provides unique predictions, part of which is potentially in line with recently reported activity patterns of neurons in the globus pallidus externus on the indirect pathway.

## Existing Hypotheses: the OpAL Model and the CS-TD Model

**FIGURE 1:**
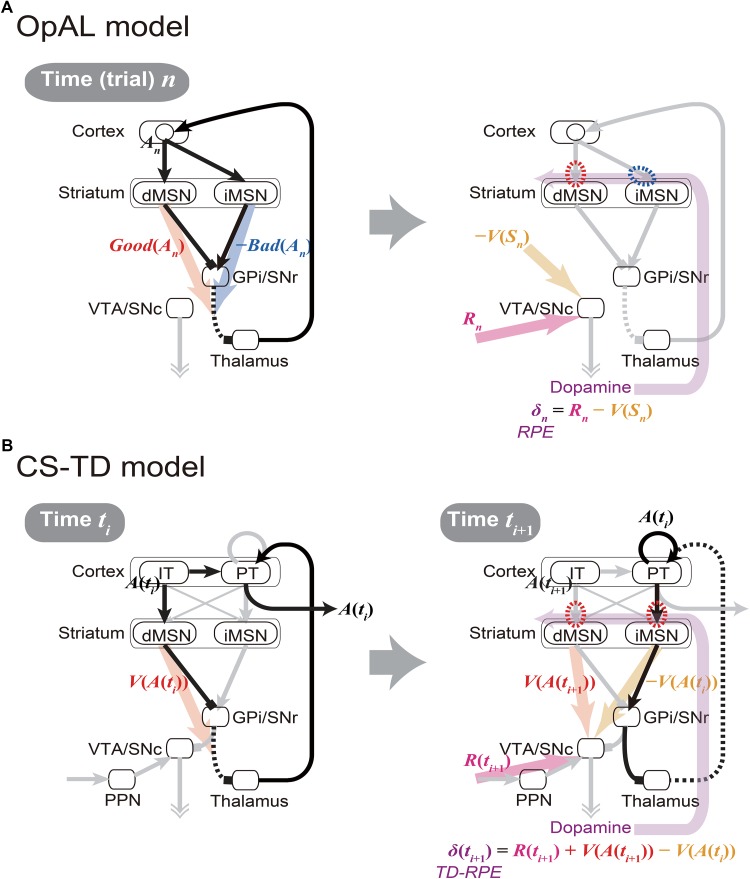
Existing models of the cortico-basal ganglia circuit functions. **(A)** Our sketch of the Opponent Actor Learning (OpAL) model (Collins and Frank, 2014), using our own terms and notations. (*Left panel*) At time (or trial) *n*, goodness (benefit) and badness (cost) of action *A_n_* [*Good*(*A_n_*) and *Bad*(*A_n_*)] are represented by the activities of striatal direct and indirect pathway medium spiny neurons (dMSNs and iMSNs), respectively. When there are multiple action candidates, one action is selected based on the utility: *Good*(*A_n_*) -*Bad*(*A_n_*), in a soft-max manner. More precisely, in the OpAL model, corticostriatal synaptic weights into dMSNs and iMSNs are defined as Go and NoGo weights (*G* and *N*), respectively, and activations of dMSNs and iMSNs are considered to be *β_G_G* and *β_N_N*, where *β_G_* and *β_N_* are parameters varying depending on DA (see Collins and Frank, 2014 for details): *Good*(*A_n_*) and *Bad*(*A_n_*) above correspond to *β_G_G* and *β_N_N*, respectively. (*Right panel*) As an outcome of action *A_n_*, reward *R_n_* is obtained, and reward prediction error (RPE): *δ_n_* = *R_n_* -*V*(*S_n_*) is represented by the dopamine (DA) neurons, where *V*(*S_n_*) is the value of state *S_n_*. When RPE is positive, the cortex-dMSN connections are potentiated (red dashed oval) whereas the cortex-iMSN connections are depressed (blue dashed oval). These contrasting plasticity inductions in turn lead to the opponent representations of goodness (benefit) and badness (cost) by dMSNs and iMSNs, respectively. Notably, there are aspects of this model that are not illustrated here; please refer to the original literature (Collins and Frank, 2014). **(B)** The Cortico-Striatal-Temporal-Difference (CS-TD) model (Morita et al., 2012; Morita, 2014). (*Left panel*) At time *t_i_*, action *A*(*t_i_*) is represented in the cortical intratelencephalic (IT) neurons, and its value [*V*(*A*(*t_i_*))] is represented by dMSNs. The information of action is transmitted to the cortical pyramidal-tract (PT) neurons, through the unidirectional IT→PT connections and also through the output nuclei of the basal ganglia [the substantia nigra pars reticulata (SNr) and the globus pallidus internus (GPi)] and the thalamus, and one action is selected in a soft-max manner when there are multiple action candidates. The action is then executed through the pyramidal tract. (*Right panel*) At time *t_i_*_+1_, PT neurons sustain the information of the executed action *A*(*t_i_*) via facilitatory recurrent excitation, and activate iMSNs via facilitatory connections so that iMSNs represent the value of the executed action [*V*(*A*(*t_i_*))]. Meanwhile, dMSNs represent the value of the upcoming action [*V*(*A*(*t_i_*_+1_))], in the same way as at time *t_i_*. The DA neurons receive positive and negative impacts from dMSNs and iMSNs, respectively, through the SNr→SNc connections. The DA neurons also receive the information of the obtained reward *R*(*t_i_*_+1_) through the pedunculopontine tegmental nucleus (PPN), and thereby calculate the temporal difference (TD) RPE: *δ*(*t_i_*_+1_) = *R*(*t_i_*_+1_) + *V*(*A*(*t_i_*_+1_)) - *V*(*A*(*t_i_*)). When TD-RPE is positive, the IT-dMSN connections and the PT-iMSN connections are both potentiated (red dashed ovals). These plasticity inductions in the same direction in turn lead to the parallel representations of action value, albeit with temporal difference, by dMSNs and iMSNs.

The cortico-basal ganglia circuits have been suggested to be crucially involved in value-related cognitive and affective processes. A prevailing hypothesis, named the Opponent Actor Learning (OpAL) model (Collins and Frank, 2014) (Figure 1A), posits that the direct and indirect pathways of the basal ganglia encode the goodness (or benefit) and badness (or cost) of options, respectively. This model, rooted in previous models (Frank et al., 2004; Frank, 2005), is based on the experimental findings indicating that the striatal direct and indirect-pathway medium spiny neurons (dMSNs and iMSNs) are positively and negatively modulated by dopamine (DA), respectively, in terms of both instantaneous responsiveness and long-term synaptic plasticity (Gerfen and Surmeier, 2011) (Figure 1A right, red and blue dashed ovals). The OpAL model explains both choice-related phenomena, such as why stimulation of dMSNs or iMSNs causes appetitive or aversive response, respectively (Kravitz et al., 2012), and motivation/effort-related phenomena, such as why DA depletion causes a shift in the preference from high-cost-high-benefit to low-cost-low-benefit options (Salamone and Correa, 2002) (i.e., according to the OpAL model, it is because dMSN’s benefit representation is weakened while iMSN’s cost representation is exaggerated) (Collins and Frank, 2014). A recent study (Kim et al., 2017) found that visually responsive neurons in the globus pallidus externus (GPe), in the middle of the indirect pathway, were largely more inhibited by objects that were stably associated with bad outcomes than by objects associated with good outcomes, suggesting that the indirect pathway signals the badness of stimuli. More recent work has further revealed that iMSNs tend to show higher activity following the presentation of lower-value conditional stimulus (Shin et al., 2018) or in response to lower-value outcome-instructing stimulus (Nonomura et al., 2018) than the case of higher-value stimulus. The OpAL model appears to be in line with these findings.

While having the strong explanatory power, however, the OpAL model still has limitations, both structurally and functionally. Specifically, at the structural level, the OpAL model, as well as most previous models, does not incorporate differentiation of two types of cortical inputs to the basal-ganglia pathways received from two types of corticostriatal pyramidal cells, namely, intratelencephalic (IT) and pyramidal-tract (PT) neurons (Cowan and Wilson, 1994; Reiner et al., 2010; Shepherd, 2013). At the functional level, the OpAL model assumes that DA represents reward prediction error (RPE) (Montague et al., 1996; Schultz et al., 1997) and induces plasticity (Reynolds et al., 2001) so as to implement value-update, but does not describe how the DA neurons calculate RPE. Also, the RPE assumed in the OpAL model takes a simple form: *R*(*t_i_*_+1_) -*V*(*t_i_*), where *R*(*t_i_*_+1_) is the obtained reward and *V*(*t_i_*) is the expected reward, whereas the experimental results have suggested that DA generally represents a more complex form of RPE called the temporal difference (TD) RPE: *R*(*t_i_*_+1_) + *V*(*t_i_*_+1_) -*V*(*t_i_*), where the additional term *V*(*t_i_*_+1_) represents the future reward(s) expected as outcome of the current/upcoming state or action, which explains the famous DA response to reward-predicting stimuli (Montague et al., 1996; Schultz et al., 1997) (see Niv and Schoenbaum (2008) for the difference between these two forms of RPE). Accordingly, the OpAL model does not describe fine temporal patterns of DA signals or MSN activity. Moreover, how the weights of synapses on dMSNs and iMSNs can converge to values corresponding to the goodness and badness of one single option (action) has actually not been shown, as pointed out by recent work (Bogacz, 2017).

In fact, there is a different hypothesis on the cortico-basal ganglia circuit functions named the Cortico-Striatal-Temporal-Difference (CS-TD) model (Morita et al., 2012, 2013; Morita, 2014; Morita and Kawaguchi, 2015) (Figure 1B), which posits that the direct and indirect pathways of the basal ganglia encode the value of the current and previous states/actions, respectively, and positively and negatively impact the DA neurons so that the temporal difference of values, i.e., *V*(*t_i_*_+1_) -*V*(*t_i_*) which constitutes the TD-RPE, can be calculated. This model is based on the experimental findings that (i) dMSNs and iMSNs are predominantly targeted by the different types of corticostriatal neurons, specifically, the IT and PT neurons, respectively (Lei et al., 2004; Reiner et al., 2010; Deng et al., 2015), (ii) IT neurons uni-directionally project to PT neurons (Morishima and Kawaguchi, 2006), which have strong facilitatory recurrent excitation (Morishima et al., 2011) that might enable sustained activity, and (iii) the output nucleus of the basal ganglia has strong inhibitory influence on the DA neurons (Tepper et al., 1995; Tepper and Lee, 2007). Although the anatomically suggested preferences in the corticostriatal connections were not supported by physiological (Ballion et al., 2008) and optogenetic (Kress et al., 2013) studies, they were supported by model fitting of short-term plasticity data (Morita, 2014), which suggested facilitatory IT→dMSN and PT→iMSN connections and depressive IT→iMSN and PT→dMSN connections.

However, the CS-TD model has a critical drawback. Specifically, although there are experimental results suggesting that DA modulates synaptic plasticity to the opposite directions in dMSNs and iMSNs (Shen et al., 2008; Gerfen and Surmeier, 2011) as the OpAL model assumes, the CS-TD model assumes the same direction of plasticity induction in dMSNs and iMSNs (Figure 1B right, red dashed ovals). As a result, the stronger inhibition of GPe neurons by bad objects (Kim et al., 2017), as well as the higher activity of iMSNs in the case of lower-value stimulus (Nonomura et al., 2018; Shin et al., 2018), cannot be explained by the CS-TD model.

## A New Hypothesis That Integrates the OpAL and CS-TD Models: the OTD Model

**FIGURE 2:**
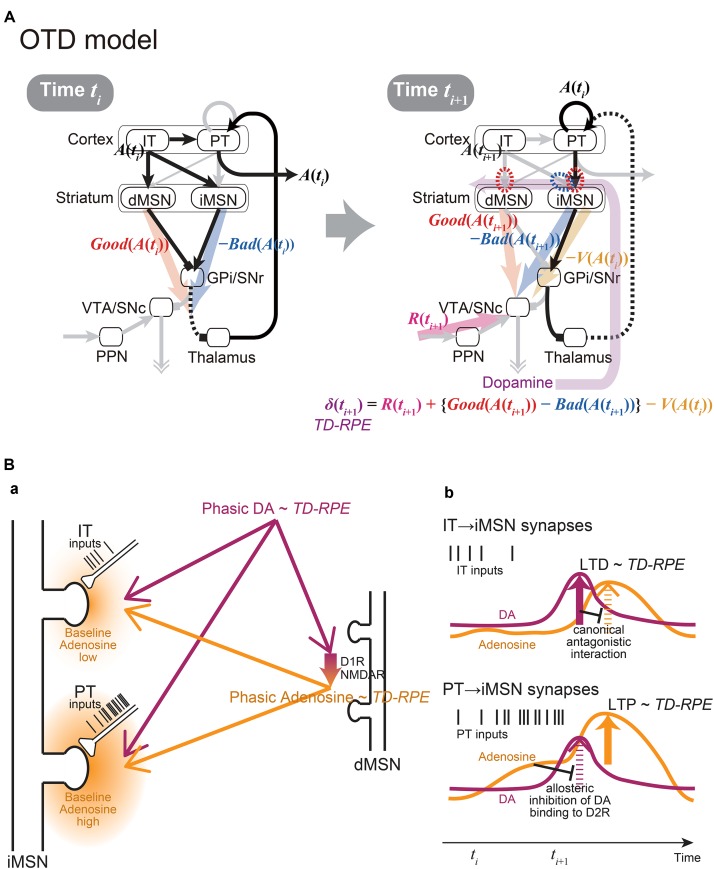
The integrated Opponency and Temporal-Difference (OTD) model, and the hypothetical mechanism for opposite directions of plasticity at IT→iMSN synapses and PT→iMSN synapses upon phasic DA release. **(A)** The OTD model. See the main text for explanation. **(B)** The hypothetical mechanism for opposite directions of plasticity at IT→iMSN synapses and PT→iMSN synapses. **(a)** A schematic diagram. The PT inputs are presumably more sustained and intense than the IT inputs, resulting in low and high baseline adenosine levels around the IT→iMSN synapses and PT→iMSN synapses, respectively. PT axospinous terminals on MSNs have been shown to be typically larger than IT axospinous terminals (Reiner et al., 2003; Reiner et al., 2010), as illustrated here, although IT axospinous terminals on iMSNs are larger than those on dMSNs (Deng et al., 2015). Phasically released DA that represents TD-RPE reaches both types of synapses similarly, while at the same time, it causes phasic adenosine release, which also reflects TD-RPE, via D1 and NMDA receptors on dMSNs. **(b)** Hypothesized time courses of DA (purple lines) and adenosine (orange lines) at IT→iMSN synapses (top panel) and PT→iMSN synapses (bottom panel). At IT→iMSN synapses, where the baseline adenosine level is low, phasic DA causes D2 receptor signaling, leading to LTD whose magnitude is proportional to TD-RPE. The D2 receptor signaling then inhibits A2A receptor signaling in response to phasic adenosine through canonical antagonistic interaction at the level of adenylyl cyclase. In contrast, at PT→iMSN synapses, high concentration of baseline adenosine allosterically prevents D2 receptor signaling to occur in response to phasic DA. Then, A2A receptor signaling occurs in response to phasic adenosine, leading to LTP whose magnitude is proportional to TD-RPE.

At first glance, these two models are mutually exclusive, because they made such contrasting assumptions on the synaptic plasticity on iMSNs. However, given that there exist two populations of corticostriatal neurons, i.e., IT and PT neurons, those assumptions might not be mutually exclusive. Specifically, if the iMSN synapses considered in the OpAL model are those targeted by IT neurons while the iMSN synapses considered in the CS-TD model are, as originally assumed, primarily PT neuron-targeting synapses, the two assumptions could go together (Figure 2A).

Crucially, the IT→iMSN connections and PT→iMSN connections are expected to have different activation profiles. In particular, because PT neurons receive uni-directional projections from IT neurons (Morishima and Kawaguchi, 2006) and excite each other via strong excitatory synapses exhibiting short-term facilitation (Morishima et al., 2011), activation of PT→iMSN synapses is expected to be delayed from, and more sustained and intense than, activation of IT→iMSN synapses (schematically illustrated by spike trains of IT and PT inputs in Figures 2Ba,b). The suggestion from model fitting (Morita, 2014) that IT→iMSN synapses and PT→iMSN synapses entail short-term depression and facilitation, respectively, can also contribute to this differentiation. At PT→iMSN synapses, such sustained intense (and facilitatory) PT inputs might generate high concentration of adenosine around the synapses, because adenosine is suggested to be released depending on glutamate receptor activation in the striatum (Pajski and Venton, 2010). Then, given the suggested allosteric inhibition of DA signaling by adenosine at A2A-D2 receptors-heteromer (Ferre et al., 1991; Ferré et al., 2018), phasic DA representing positive RPE is expected not to be able to induce long-term depression (LTD) through D2 receptor (D2R) signaling. Moreover, given that DA is suggested to cause adenosine release through activations of D1 receptors (D1Rs) and NMDA receptors in the nucleus accumbens (Harvey and Lacey, 1997; Wang et al., 2012), we assume that the RPE-representing phasic DA induces phasic adenosine that also reflects RPE: since adenosine causes vasodilation (Phillis, 1989) presumably on a sub-second time scale (Wang and Venton, 2017), such RPE-reflecting phasic adenosine may cause oxygen changes that could underlie the widely reported striatal fMRI-BOLD signals correlated with RPE (McClure et al., 2003; O’Doherty et al., 2003). The positive RPE-representing phasic adenosine is then expected to induce long-term potentiation (LTP) of PT→iMSN synapses through A2A receptor signaling (c.f., Shen et al., 2008) (Figure 2B). In contrast, at IT→iMSN synapses where adenosine is scarce, phasic DA representing positive RPE is assumed to cause LTD via D2R signaling, which could then inhibit A2A receptor signaling through the suggested canonical antagonistic interaction at the level of adenylyl cyclase (Kull et al., 1999; Hillion et al., 2002; Navarro et al., 2014; Ferré et al., 2018).

**FIGURE 3:**
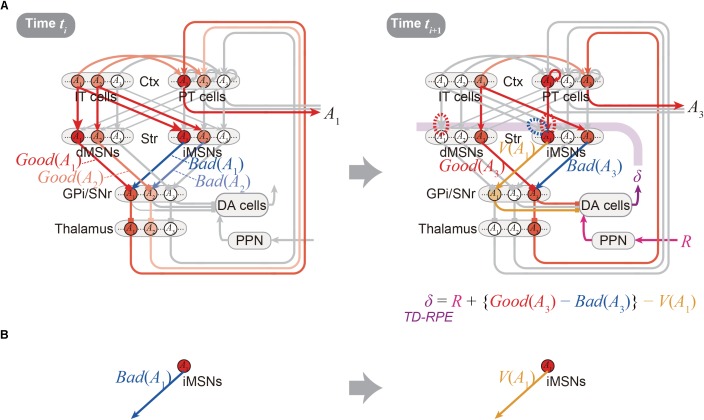
Detailed operation of the OTD model, and reversal of the valence in the coding of the indirect pathway predicted by the model. **(A)** Detailed operation of the OTD model. (*Left panel*) At time *t_i_*, goodness (benefit) and badness (cost) of each of the two action candidates, *A*_1_ and *A*_2_, are represented in the direct and indirect pathways, respectively. Based on the utility combining those benefit and cost, one action, *A*_1_, is selected in a soft-max manner to be represented by a population of PT neurons, and executed through the pyramidal tract. (*Right panel*) At time *t_i_*_+1_ when reward comes as an outcome of the executed action *A*_1_, the *A*_1_-corresponding population of PT neurons sustain their activity, activating the *A*_1_-corresponding population of iMSNs. These iMSNs represent the value of the executed action [*V*(*A*_1_)], and negatively impact the DA neurons via GPi/SNr. In the meantime, goodness (benefit) and badness (cost) of the upcoming action *A*_3_, i.e., *Good*(*A*_3_) and *Bad*(*A*_3_) are represented in the *A*_3_-corresponding populations of dMSNs and iMSNs, respectively, which positively and negatively impact the DA neurons. Together with reward-representing input *R*, the DA neurons calculate a form of TD-RPE: *R* + {*Good*(*A*_3_) - *Bad*(*A*_3_)} - *V*(*A*_1_) [results of recent work (Kim et al., 2015) imply that DA neurons involved in learning of stable values do not receive reward-representing input *R*; they may represent TD error: *Good*(*A*(*t_i_*_+1_)) -*Bad*(*A*(*t_i_*_+1_)) -*V*(*A*(*t_i_*))]. When this TD-RPE/TD-error is positive, the *A*_1_-corresponding IT-dMSN connections and IT-iMSN connections are potentiated and depressed, respectively, and the *A*_1_-corresponding PT-iMSN connections are potentiated. These differential plasticity inductions depending on both cortical and striatal neuron types in turn lead to the representations of benefit, cost, and action value by each pathway. **(B)** The OTD model predicts a reversal of the bad–good valence in the coding of the indirect pathway: the *A*_1_-corresponding iMSN initially represents the badness (cost) of *A*_1_ (left) but later represents the value (≈ goodness – badness) of the same *A*_1_ (right).

Figure 2A shows the integrated Opponency and Temporal-Difference (OTD) model. At time *t_i_* (Figure 2A, left), action *A*(*t_i_*) is represented by a population of cortical IT neurons, and its goodness (benefit) and badness (cost) [*Good*(*A*(*t_i_*)) and *Bad*(*A*(*t_i_*))] are represented by dMSNs and iMSNs, respectively, so that the utility of the action, i.e., *Good*(*A*(*t_i_*)) -*Bad*(*A*(*t_i_*)) is computed in the downstream. When there are multiple action candidates, one action is selected based on the utility in a soft-max manner. The selected action is represented by the cortical PT neurons, which are driven by the IT neurons and the basal ganglia output, and executed through the pyramidal tract. At time *t_i_*_+1_ (Figure 2A, right), a population of dMSNs and a population of iMSNs represent the goodness (benefit) and badness (cost) of the upcoming action [*Good*(*A*(*t_i_*_+1_)) and *Bad*(*A*(*t_i_*_+1_))], respectively, while a different population of iMSNs represents the value of the executed action [*V*(*A*(*t_i_*))]. The dMSN population and iMSN populations positively and negatively modulate the DA neurons via the basal ganglia output, respectively, so that the DA neurons compute a form of TD-RPE: *δ*(*t_i_*_+1_) = *R*(*t_i_*_+1_) + {*Good*(*A*(*t_i_*_+1_)) -*Bad*(*A*(*t_i_*_+1_))}-*V*(*A*(*t_i_*)). When the TD-RPE is positive, the IT-dMSN connections are potentiated (red dashed oval in Figure 2A right) whereas the IT-iMSN connections are depressed (blue dashed oval), and the PT-iMSN connections are potentiated (red dashed oval). Figure 3A shows the operation of the OTD model in more detail, illustrating different populations of neurons corresponding to different actions. Notably, the IT/PT-iMSN connections corresponding to the previous action that constitutes a cause of the TD-RPE (action “*A*_1_” in the figure) are plastically changed whereas the IT/PT-iMSN connections corresponding to the current action (“*A*_3_” in the figure) are not, ensuring the causality; this could be achieved through mechanisms for creating a delayed time window for plasticity, such as those revealed for the synapses on dMSNs (Yagishita et al., 2014). As shown in Figures 2A and 3A, the OTD model literally has functions of both OpAL and CS-TD models. Specifically, the direct and indirect pathways serve for good-bad(benefit-cost)-analysis of current states/actions/options, and simultaneously perform the calculation of TD-RPE, which is used for updating the value of previous states/actions/options. This is enabled by the duality of the role of iMSNs: initially representing the badness (cost) of a state/action/option and later representing the value (≈ goodness – badness) of the same state/action/option (Figure 3B).

## Predictions, Limitations, and Perspectives

The OTD model provides testable predictions, a few of which will be described below. First, since iMSNs are assumed to initially represent the badness and later represent the overall value as mentioned just above, a reversal of the valence in the coding of the indirect pathway is predicted to be likely to occur (Figure 3B). This is potentially in line with a result reported in a recent study, which examined the response of visually responsive GPe neurons, on the indirect pathway, to objects that were stably associated with good or bad outcomes (Kim et al., 2017). These GPe neurons are largely more inhibited by the presentation of bad objects, consistent with the iMSN’s coding of badness assumed in the OpAL or OTD models. But later on, the value-coding responses were reversed, on average, so that these neurons became more inhibited, albeit slightly, by good objects (Figure 4C of Kim et al., 2017). This is potentially in line with the OTD model’s operation, although the observed reversal could instead reflect a similar reversal in the DA neuronal activity (Figure 3E bottom of Kim et al., 2015) via modulations of iMSNs’ activity by DA. The predicted reversal of the valence of value-coding in the indirect pathway in the OTD model could also explain why good-preferring neurons outnumbered bad-preferring neurons in the striatum (Kim and Hikosaka, 2013) while dMSNs and iMSNs are roughly equinumerous, a point raised in a recent review (Hikosaka et al., 2018). The second prediction of the OTD model is that the activity of IT→dMSN/IT→iMSN pathways representing the goodness/badness not only biases current choice but also contributes to DA signal representing TD-RPE used for updating the value of previous state/action and thereby biases future choices. This is potentially in line with the recently suggested role of iMSNs in lose-switch, i.e., choice switching following bad outcomes (Nonomura et al., 2018). Moreover, if these pathways entail differential short-term plasticity as predicted by model-fitting (Morita, 2014), i.e., facilitation at IT→dMSN and depression at IT→iMSN, DA neurons could receive biphasic impacts, i.e., initially negative impact via the indirect pathway and subsequently positive impact via the direct pathway. Then, a recently proposed mechanism (Bogacz, 2017) might enable TD (higher-order) learning of both goodness and badness of one single option (action).

The OTD model also has limitations. The model’s key assumption lies in the plasticity of corticostriatal synapses depending on DA and adenosine. Regarding this topic, recent work (Fisher et al., 2017) has shown that, in both putative dMSNs and iMSNs, repetition of “pre-post” activity paring followed by reward-predicting sensory inputs causes potentiation of response to contralateral cortical stimulation, which presumably activates IT axons (because IT cells, but not PT cells, project to the contralateral cortex/striatum; Cowan and Wilson, 1994). This is apparently not in line with any of the OTD, OpAL, or CS-TD models. However, they have also shown results indicating that blockade of adenosine A2A receptors changes potentiation in iMSNs into depression. Considering this, a conceivable possibility is that, in their experiment, electrical stimulation of IT axons resulted in richer adenosine around IT→iMSN synapses than the natural condition (i.e., to the level comparable to, or even beyond, the PT→iMSN synapses in the natural condition), leading to potentiation of IT→iMSN synapses that would naturally undergo depression. It should also be noted that the authors (Fisher et al., 2017) described that in their protocol ”*adenosine signaling is also likely to be coincident with light flash evoked dopamine signaling* (p. 10)”; our assumption that phasic DA induces phasic adenosine would be consistent with this argument.

Another recent work (Yapo et al., 2017) examined the effects of transient (rather than tonic) DA inputs, with or without tonic adenosine (agonist) inputs, on the intracellular signaling in both D1 and D2R-expressing cells (presumably dMSNs and iMSNs, respectively) by using DA uncaging. It found (Yapo et al., 2017) that, under the presence of tonic adenosine input in D2-MSNs, transient DA input causes a reduction in cAMP, but its efficacy is similar to the efficacy of DA-dependent cAMP increase in D1-MSNs, challenging the traditional notion that D2R signaling is much more effective than D1R signaling. Moreover, at the downstream of cAMP, transient DA (with tonic adenosine) hardly decreased the level of PKA-dependent phosphorylation (Yapo et al., 2017). Counteraction of D2R signaling by A2AR stimulation has also been shown in previous studies with bath application of D2R agonist (Azdad et al., 2009; Higley and Sabatini, 2010). These could potentially support the OTD model’s impaired D2R signaling at adenosine-rich PT→iMSN synapses, although the authors of the abovementioned recent study (Yapo et al., 2017) suggested that allosteric inhibition of D2R signaling by adenosine may not be included, different from our assumption. The same study (Yapo et al., 2017) further indicated, through mathematical modeling based on the previous work (Nair et al., 2015), that D2-MSNs would also have a different, “tone-sensing” mode, in which phasic DA reduction effectively causes PKA-dependent phosphorylation. This mode was achieved by assuming high tonic DA in their simulations, but the authors discussed that the switch between the different modes may also result from changes in adenosine. The OTD model’s adenosine-level-dependent differential plasticity between IT→iMSN and PT→iMSN synapses is potentially in line with their discussion.

Yet another important experimental result regarding adenosine is that striatum-specific knockout of A2A receptors caused selective impairment of habit formation (Yu et al., 2009). This is also hard to explain by the OTD, OpAL, or CS-TD models. One possibility is that there exist several (or many) mechanisms for TD-RPE calculation and the OTD model is just one of them specifically operating in the dorsal striatum, where adenosine release evoked by stimulation was robustly detected (Pajski and Venton, 2010), while other mechanisms, e.g., those involving striosomes, operate in more ventral parts of the striatum. Existence of multiple mechanisms for TD-RPE calculation seems to be in line with the observed distributed RPE-related information in the regions projecting to DA neurons (Tian et al., 2016). Then, knockout of A2A receptors might particularly impair the learning function of the dorsal striatum, which, or more specifically the dorsolateral striatum, is suggested to be crucial for habit formation (Everitt and Robbins, 2005; Burton et al., 2015). In addition to the issues so far described, there are important issues that need to be addressed so as to validate, deny, or elaborate the OTD model (Box 1).

**BOX 1. Outstanding issues.**

**Differences Between IT→iMSN Synapses and PT→iMSN Synapses**

– The OTD model assumes that sustained intense PT inputs generate rich adenosine so that the local baseline adenosine concentration around PT→iMSN synapses is higher than the concentration around IT→iMSN synapses. Does such local regional variation indeed exist?

– It has been shown that PT-type axospinous synaptic terminals on MSNs are typically larger than IT-type axospinous synaptic terminals (Reiner et al., 2003; Reiner et al., 2010), although IT axospinous terminals on iMSNs are larger than those on dMSNs (Deng et al., 2015). Does the size difference between IT and PT axospinous terminals also relate to the hypothesized differential basal adenosine levels and/or plasticity inductions between IT→iMSN synapses and PT→iMSN synapses?

– Do the A2A receptors exist at/around IT→iMSN synapses and PT→iMSN synapses equally or differentially? Ultrastructural immunohistochemical study examining rat striatum (Hettinger et al., 2001) observed A2AR immunoreactivity primarily at asymmetric (putative excitatory) synapses and less frequently at symmetric (putative inhibitory) synapses, but whether A2ARs are differentially distributed among different types of excitatory synapses receiving IT, PT, and thalamic inputs remains to be seen.

**DA-Dependent Adenosine Release**

– DA-dependent adenosine release was indicated in the nucleus accumbens *in vitro* (Harvey and Lacey, 1997; Wang et al., 2012). Does similar release occur also in the dorsal striatum *in vivo*? What are the time and spatial scales of the DA-dependent adenosine release? Looking at Fig. 5B of (Wang et al., 2012), it seems that the effect of D1R agonist SKF38393 on the paired-pulse ratio of cortico-D1-MSN transmission, which was suggested to be mediated by adenosine, began to appear soon after the application of agonist, although the exact latency is difficult to read out. It thus seems not impossible that DA-dependent adenosine release occurs in a fast time scale, but this issue, as well as the spatial spread of released adenosine (in particular, whether it can affect synaptic plasticity in iMSNs), needs to be experimentally examined with high temporal/spatial resolutions.

– If adenosine release is indeed induced by phasic DA that signals TD-RPE, can the concentration of adenosine also reflect TD-RPE? Reward-related oxygen changes in the rat nucleus accumbens have been observed and suggested to be consistent with RPE-representing fMRI-BOLD signals in humans (Francois et al., 2012). Given that adenosine causes vasodilation (Phillis, 1989; Wang and Venton, 2017), it seems conceivable that DA-dependent release of adenosine contributes to such oxygen changes, and this would be interesting to examine.

**Plasticity**

– Do the hypothesized differential DA and adenosine-dependent plasticity inductions at IT→iMSN and PT→iMSN synapses indeed occur? Since experimental validation would not be straightforward, it would be desired to construct mathematical models, based on previous models of the signaling cascades in MSNs (Lindskog et al., 2006; Nakano et al., 2010; Nair et al., 2015). Known properties of adenosine (Schiffmann et al., 2007; Wall and Dale, 2008; Ferré et al., 2018), time course of phasic DA release (Day et al., 2007; Yagishita et al., 2014; Nair et al., 2016), and also dendritic morphology (Lindroos et al., 2018) and spines (Blackwell et al., 2018) are desired to be incorporated. Moreover, because adenosine, as well as DA, has been shown to modulate not only synaptic plasticity but also synaptic transmission (Shindou et al., 2008), such effects are also desired to be incorporated in future models.

– We assumed that, at IT→iMSN synapses, phasic DA representing positive TD-RPE causes LTD via D2R signaling in iMSNs. However, recent work conducting cell-type-specific removal of D2R (Augustin et al., 2018) has shown, using high-frequency stimulation for LTD induction (Calabresi et al., 1992), that D2R signaling in iMSNs only weakly modulates LTD in iMSNs while D2R signaling in cholinergic interneurons strongly modulates LTD in both dMSNs and iMSNs. Given this, the assumed positive TD-RPE-dependent LTD at IT→iMSN synapses might actually occur through D2R signaling not in iMSNs but in cholinergic interneurons, while the same LTD induction at PT→iMSN synapses could be masked by adenosine-dependent LTP. Instead, decay/forgetting (c.f., Morita and Kato, 2014; Kato and Morita, 2016) and/or homeostatic plasticity could operate as a functional alternative to LTD.

– What occurs when TD-RPE is negative? Negative TD-RPE-representing phasic decrease in DA would drastically shift the balance of D2R/A2AR signaling to the A2AR side so as to induce LTP. For the OTD model to hold also when TD-RPE is negative, however, it would be desired that whereas IT→iMSN synapses undergo LTP, PT→iMSN synapses do not (and rather undergo LTD). Whether and how such differentiation between IT→iMSN synapses and PT→iMSN synapses can arise remain to be examined. There is a recent finding that is possibly related to this. Specifically, impairment in LTP induction in A2R-expressing MSNs (i.e., iMSNs) was observed in *Rhes* (a GTPase enriched in MSNs) knockout female mice, and it was indicated to be associated to excessive phasic cAMP/PKA signaling (Ghiglieri et al., 2015). In light of this result, we speculate that when TD-RPE is negative and DA phasically decreases, at IT→iMSN synapses, moderate A2AR/cAMP signaling leads to LTP induction, whereas at PT→iMSN synapses where PT inputs generate high baseline adenosine, excessive A2AR/cAMP signaling prevents LTP induction.

– At the algorithm level, what plasticity rules can ensure that the weights of IT-dMSN synapses, IT-iMSN synapses, and PT-iMSN synapses converge to the goodness, badness, and action-value, respectively?

**Circuit Connectivity**

– Whereas the CS-TD model assumed preferential IT→dMSN and PT→iMSN transmissions, the OTD model no longer assumes IT→dMSN preference given that the IT→iMSN connections are now assumed to encode the badness of current option. However, the situation remains elusive for PT→dMSN/iMSN connections. One possibility, extending the OTD model, is that the PT→iMSN connections and PT→dMSN connections represent the goodness and badness of the executed action, respectively.

– The OTD (or CS-TD) model assumes that activation of dMSNs and iMSNs has net positive and negative impacts on the activity of DA neurons (or DA release), respectively. Potentially in line with this, stimulation of the terminals of nucleus-accumbens D1R-MSNs led to disinhibition of DA neurons in the ventral tegmental area (Bocklisch et al., 2013; Keiflin and Janak, 2015). Also, stimulation of caudate tail caused a phasic increase of activity in a population of DA neurons, possibly through the substantia nigra pars reticulata (SNr) (Kim et al., 2015). Regarding the indirect pathway, chemical excitation of rat GP (homologous to primate GPe) resulted in an elevation in neostriatal DA levels presumably disynaptically via SNr (Lee et al., 2004). However, this last study indicated that the increase in DA release was due to an increase in burst firing rather than in firing rate. Whether changes in firing rate can occur remains to be seen, while extension of the OTD model to incorporate temporal coding beyond firing rate will also be an important future direction.

**Consistency With *In Vivo* Experimental Results**

– The OTD (or CS-TD) model assumes that PT neurons can sustain activity via strong facilitatory recurrent excitation (Morishima et al., 2011). This point has been challenged by a recent study (Saiki et al., 2018) showing that extratelencephalic (ET) pyramidal cells, which would largely overlap with PT neurons, exhibit post-spike suppression (i.e., suppression of the generation of a next spike with a short duration) *in vivo* and arguing that it would interrupt sustained activity. Although this is an important argument, if successive PT→PT inputs with short durations cause synaptic short-term depression, post-spike suppression could actually be beneficial for its prevention. Also related to this point, recent studies have shown that sustained activity is maintained through cortico-thalamic interactions (Bolkan et al., 2017; Guo et al., 2017; Schmitt et al., 2017). Because PT neurons, but not IT neurons, innervate thalamus, PT neurons may sustain activity through the interaction with thalamus.

– It has been shown that dMSNs and iMSNs are concurrently activated during action initiation (Cui et al., 2013). Such concurrent activation can be in line with the OpAL or OTD model, but seems difficult to explain by the CS-TD model. The OTD (or CS-TD) model, however, also predicts sustained activity of iMSNs representing previous value, which was not shown in the experiments (Cui et al., 2013). This potential discrepancy could be resolved in multiple ways. First, if goodness (benefit) and badness (cost) of an action are nearly comparable, overall value (≈ benefit – cost) is expected to be small and can be difficult to detect. Second, in the OTD model, representation of goodness and badness is transiently done for all the action candidates/options (*A*_1_ and *A*_2_ at *t_i_* in the case of Figure 3A) whereas sustained representation of previous value is done only for the single action that was actually chosen/executed (*A*_1_ at *t_i_*_+1_ in Figure 3A), and therefore the latter can be more difficult to detect than the former. Third, the goodness/badness representation and the previous-value representation could be done with different firing patterns, in particular, bursty and nonbursty firings, respectively. If so, the former can generate larger calcium transients that are easier to detect. These explanations are, however, all speculations, and direct experimental test of whether previous value is represented in iMSNs is desired.

## Author Contributions

KM conceived of the hypothesis, and elaborated it through discussion with YK.

## Conflict of Interest Statement

The authors declare that the research was conducted in the absence of any commercial or financial relationships that could be construed as a potential conflict of interest.
